# Prediction of Acute Kidney Injury by Cystatin C and [TIMP-2]*[IGFBP7] after Thoracic Aortic Surgery with Moderate Hypothermic Circulatory Arrest

**DOI:** 10.3390/jcm11041024

**Published:** 2022-02-16

**Authors:** Kevin Pilarczyk, Bernd Panholzer, Katharina Huenges, Mohamed Salem, Toni Jacob, Jochen Cremer, Assad Haneya

**Affiliations:** 1 Department of Intensive Care Medicine, Imland Klinik Rendsburg, 24768 Rendsburg, Germany; 2 Department of Cardiovascular Surgery, University of Schleswig-Holstein, 24105 Kiel, Germany; bernd.panholzer@uksh.de (B.P.); katharina.huenges@uksh.de (K.H.); mohamed.salem@uksh.de (M.S.); toni.jacob@uksh.de (T.J.); jochen.cremer@uksh.de (J.C.); assad.haneya@uksh.de (A.H.)

**Keywords:** acute kidney injury, hypothermic circulatory arrest, thoracic aortic surgery, biomarkers, Cystatin, cell cycle arrest markers

## Abstract

(1) Background: Acute kidney injury (AKI) is a common complication following thoracic aortic surgery (TAS), with moderate hypothermic circulatory arrest (MHCA). However, prediction of AKI with classical tools remains uncertain. Therefore, it was the aim of the present study to evaluate the role of new biomarkers in patients after MHCA. (2) Methods: 101 consecutive patients were prospectively enrolled. Measurements of urinary [TIMP-2]*[IGFBP7] and Cystatin C in the blood were performed perioperatively. Primary endpoint was the occurrence of AKI stage 2 or 3 (KDIGO-classification) within 48 h after surgery (AKI group). (3) Results: Mean age of patients was 69.1 ± 10.9 years, 35 patients were female (34%), and 13 patients (13%) met the primary endpoint. Patients in the AKI group had a prolonged ICU-stay (6.9 ± 7.4 days vs. 2.5 ± 3.1 days, *p* < 0.001) as well as a higher 30-day-mortality (9/28 vs. 1/74, *p* < 0.001). Preoperative serum creatinine (169.73 ± 148.97 μmol/L vs. 89.74 ± 30.04 μmol/L, *p* = 0.027) as well as Cystatin C (2.41 ± 1.54 mg/L vs. 1.13 ± 0.35 mg/L, *p* = 0.029) were higher in these patients. [TIMP-2]*[IGFBP7] increased significantly four hours after surgery (0.6 ± 0.69 mg/L vs. 0.37 ± 0.56 mg/L, *p* = 0.03) in the AKI group. Preoperative Cystatin C (AUC 0.828, *p* < 0.001) and serum creatinine (AUC 0.686, *p* = 0.002) as well as [TIMP-2]*[IGFBP7] 4 h after surgery (AUC 0.724, *p* = 0.020) were able to predict postoperative AKI. The predictive capacity of Cystatin C was superior to serum creatinine (*p* = 0.0211) (4) Conclusion: Cystatin C represents a very sensitive and specific biomarker to predict AKI in patients undergoing thoracic surgery with MHCA even before surgery, whereas the predictive capacity of [TIMP-2]*[IGFBP7] is only moderate and inferior to that of serum creatinine.

## 1. Introduction

More than 30 years ago moderate hypothermic circulatory arrest (MHCA) was introduced for cerebral protection during aortic arch surgery. Improved surgical and anesthetic techniques have facilitated increasingly complex aortic reconstructions with decreased morbidity and mortality. Although MHCA has proven to be an effective technique for protecting various organs from ischemic damage as a result of circulatory arrest, the incidence of acute kidney injury (AKI) after thoracic aortic surgery ranges from 18% to 55% being higher than in other aortic procedures [1,2,3,4,5]. Renal dysfunction leads to increased postoperative morbidity and mortality, and patients requiring renal replacement therapy (RRT) have an elevated short-term mortality of up to 64% [1,2,3,4,5].

Risk assessment for AKI is recommended by clinical practice guidelines but remains imprecise mainly due to very limited sensitivity and specificity of early diagnostic tests available for AKI. Although serum creatinine is known to be an inadequate and delayed marker of acute changes in renal function, it is currently accepted as “gold standard” for the diagnosis of AKI due to the lack of other reliable biomarkers [6]. Obviously, there is a clear need for sensitive and specific biomarkers allowing early identification of patients with high risk for AKI to initiate preventive or therapeutic interventions [7]. Cystatin C is an accurate biomarker for the early detection of AKI, and may, in selected populations, be superior to creatinine; however, data have been inconsistent and Cystatin C is modified by age, sex, muscle mass, obesity, smoking status, thyroid function, inflammation, and malignancy [8].

A biomarker test based on a combination of urine tissue inhibitor of metalloproteinases 2 (TIMP-2) and insulin-like growth factor binding protein 7 (IGFBP7) was recently approved by the US Food and Drug Administration for AKI risk stratification. TIMP-2 and IGFBP7 have been linked to cell-cycle arrest, which is known to be involved in the pathogenesis of AKI [9]. Several studies have reported that [TIMP-2]*[IGFBP7] can be used to predict AKI after cardiac surgery [10,11]. Neither Cystatin C nor [TIMP-2]*[IGFBP7] have been evaluated in the specific population of patients undergoing thoracic surgery with the use of MHCA.

The aim of this investigation was to identify and describe incidence of risk factors for AKI during thoracic aortic surgery (TAS) under MHCA and to analyze the predictive accuracy of Cystatin C and [TIMP-2]*[IGFBP7] for the development of AKI.

## 2. Materials and Methods

### 2.1. Patients (Inclusion and Exclusion Criteria)

The present study was approved by the Institutional Ethical Review Board and informed consent from the patient or the patient’s next of kin was obtained. We included all patients (minimum age: 18 years) scheduled for thoracic aortic surgery (TAS) under MHCA between December 2016 and March 2018. Patients with acute aortic dissection, preoperative renal replacement therapy and patients that died within 48 h after surgery were excluded. We used the Standards for Reporting of Diagnostic Accuracy (STARD) statement for planning and conducting the study and preparing the manuscript [12]. The study protocol was approved by the local Ethics Committee and the necessary individual patient consent was obtained during the hospital stay.

### 2.2. Surgical Procedure

All operations were carried out under general anesthesia by senior surgeons. After median sternotomy, pericardiotomy was carried out longitudinally, followed by direct cannulation of the distal ascending aorta. Cannulation of the right atrium with a common two-stage venous cannula was the standard venous drainage. An antegrade and retrograde injection of cold blood cardioplegic solution was used as a standard for myocardial protection in all cases. MHCA was performed with a core temperature of 24 ± 2 °C that was measured naso-pharyngeally. Continuous CO_2_ insufflation was used to avoid cardiac air embolism. In cases in which the hypothermic circulatory arrest had to be continued for more than 20 min, an antegrade bilateral cerebral perfusion with oxygenated cold blood (18 °C) was introduced into the head and neck vessels of the aortic arch through a balloon catheter with a pressure of 50–60 mmHg. Near infrared spectroscopy (NIRS) was used to monitor brain tissue oxygenation. Residual air in the left ventricle was removed by restarting retrograde perfusion slowly via the venous cannula beside CO_2_ insufflation. After insertion of the aortic cannula directly in the vascular graft, cardiopulmonary bypass was restarted after clamping the aortic prosthesis distally. After suturing the proximal anastomosis, a cardiac de-airing was carried out through a small incision in the aortic prosthesis before opening the clamp. Residual air in the left side of the heart was controlled by transesophageal echocardiography as described previously [13].

### 2.3. Biomarker Measurements

Urine and samples for biomarker analysis were obtained preoperatively as well as in the early postoperative course after surgery: We analyzed urinary (TIMP-2)*(IGFBP7) at four time points: within 1 h before surgery, 2 h after surgery, 4 h after surgery, and at 8.00 a.m. on postoperative day one (POD 1). In addition, we measured Cystatin C in the blood at the following time-points: within 1 h before surgery, 2 h after surgery, 6 h after surgery, and at 8.00 a.m. POD 1. These time points were chosen from recently published studies to be able to compare cut-off-values. Urine samples were centrifuged within one hour of collection using polypropylene urine collection cups. Within four hours of collection or after storage at 2–8 °C for up to 20 h, urinary TIMP-2 and IGFBP7 levels were measured with the commercially available and FDA-approved NephroCheck™ Test (Astute Medical, San Diego, CA, USA)—a point-of-care test kit developed to measure and calculate the product of [TIMP-2] and [IGFBP7] concentration in the urine. Cystatin C levels were measured in the patient’s serum based on immuno-turbidimetric assay (normal range: 0.61–0.95 mg/L). The cystatin C reagent contained a suspension of latex particles coated with purified goat anti-human cystatin C polyclonal antibodies. A sample was mixed with this suspension. The resulting immune complexes were measured by turbidimetry. The signal generated was correlated with the concentration of cystatin C in the sample. By interpolation on a standard curve, the concentration of cystatin C in the sample was calculated.

Physicians in charge were blinded for the cell cycle arrest biomarker levels and laboratory investigators were blinded to clinical outcomes. Glomerular filtration rate was calculated with the Cockcroft-Gault formula (estimated Glomerular filtration rate = eGFR).

### 2.4. Definition of Endpoint and Outcomes

AKI stage was determined daily based on diuresis rate and serum creatinine concentration according to the Kidney Disease: Improving Global Outcomes classification (KDIGO) as follows [14]:

- AKI 1: Increase of serum creatinine by ≥0.3 mg/dL (≥26.4 µmol/L) or increase to ≥150–200% from baseline or urine output <0.5 mL/kg/h for >6 h;
- AKI 2: increase of serum creatinine to >200–300% from baseline and/or urine output <0.5 mL/kg/h for >12 h;
- AKI 3: increase of serum creatinine to >300% from baseline or serum creatinine ≥4.0 mg/dL (≥354 µmol/L) after a rise of at least 44 µmol/L or treatment with renal replacement therapy and/or urine output <0.3 mL/kg/h for >24 h or anuria for 12 h.

The primary endpoint was the new occurrence of AKI stage 2 or 3 within 48 h after surgery. AKI 2 and 3 were grouped together, as previously published studies have shown that AKI 1 has only minor impact on outcome whereas AKI 2 and 3 are associated with significantly increased postoperative morbidity and mortality. The observation period of 48 h was chosen because prior studies have shown that AKI occurs within the first 24–72 h after surgery in the majority of cases. Secondary endpoints included AKI at any stage as well as AKI with need for renal replacement therapy.

### 2.5. Sample Size Calculation

The aim of the present study was to prove the hypothesis that mean urine [TIMP-2]*[IGFBP7] levels and Cystatin C levels in patients with AKI differ significantly to those of patients without AKI within the first 24 h after surgery. Therefore, a tailed power analysis based on the available published data on [TIMP-2]*[IGFBP7] in the adult cardiothoracic population was performed to calculate the sample size. We aimed to detect a difference of one unit in [TIMP-2]*[IGFBP7] levels with a standard deviation of 0.8 units [10]. Expected incidence of AKI 2–3 was 10%. With a given probability of type I error (α) of 0.05, and a power (1 − β) of 0.9, sample size calculation revealed a required minimum size of 60 patients in total.

### 2.6. Statistical Analysis

Statistical analyses were performed with SPSS Statistics 19 (IBM, Chicago, IL, USA). Continuous data were expressed as mean ± SD; categorical data were expressed as percentage. Comparisons between two groups were carried out using unpaired Student’s t-test for normally distributed data, or the Mann-Whitney Rank Sum Test for non-normally distributed data. Multiple groups were compared with ANOVA. Statistical significance was assumed for a *p*-value < 0.05. To measure the sensitivity and specificity of urinary [TIMP-2]*[IGFBP7] at different cut-off values, a conventional receiver operating characteristic (ROC) curve was generated.

## 3. Results

### 3.1. Patients’ Characteristics

**Figure 1:**
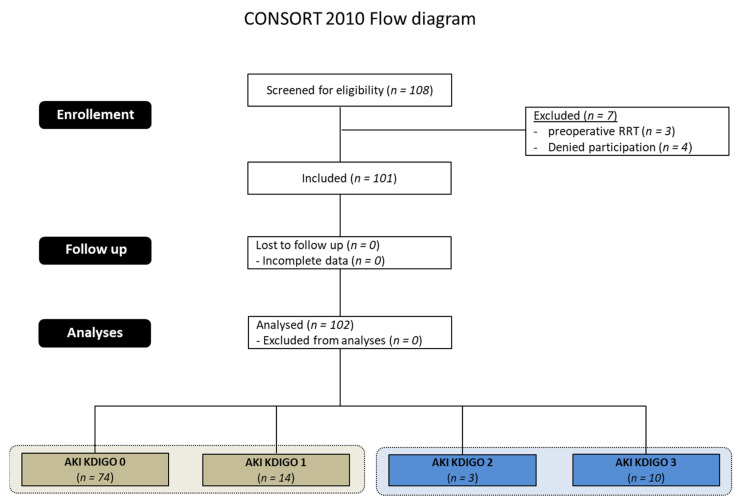
CONSORT (Consolidated Standards of reporting trials) 2010 Flow diagram. AKI = acute kidney injury, KDIGO = Kidney Disease: Improving Global Outcomes, RRT = replacement therapy, RRT = Renal replacement Therapy.

101 patients undergoing TAS with MHCA were included in this study. 27 patients (26%) developed AKI of any stage within 48 h after surgery: 14 patients (14%) developed mild AKI. AKI was classified as AKI 2 or 3 in 13 patients (14%), and 11 patients (11%) required renal replacement therapy (RRT) (Figure 1).

The distribution of applied criteria for AKI staging on the basis of diuresis and serum creatinine are given in Table 1: 11 patients were defined as AKI primarily on the basis of increased serum creatinine concentration, and 16 patients reached the threshold for a low diuresis rate for the first time.

Pre-Procedural as well as operative variables and postoperative outcomes of patients suffering from AKI stage 2 or 3 compared to those without significant postoperative dysfunction (AKI KDIGO 0 or 1) are summarized in Table 2. Patients with renal impairment were significantly older than those without (72.72 ± 6.19 years vs. 68.51 ± 11.46 years, *p* = 0.044). The EuroScore predicting perioperative mortality was significantly higher in the AKI group (ES log: 29.68 ± 19.98 vs. 19.89 ± 14.66, *p* = 0.026, ES II: 16.29 ± 11.49 vs. 5.73 ± 5.25, *p* = 0.003, ES I: 13.15 ± 6.09 vs. 9.99 ± 4.55, *p* = 0.020). Incidence of CKD (5/13 (38.5%) vs. 0/88 (0%), *p* = 0.001), as well as the proportion of patients undergoing non-elective procedures (5/13 (38.5%) vs. 15/88 (17.0%), *p* = 0.02), was higher in patients with postoperative kidney injury.

In addition, length of surgery and length of cardiopulmonary bypass were longer in the AKI group (397.20 ± 139.04 min vs. 297.77 ± 93.29 min, *p* = 0.017, 230.21 min ± 68.29 vs. 170.57 ± 51.05 min, *p* = 0.007).

Intraoperative diuresis was lower (557.14 ± 605.06 mL vs. 989.82 ± 788.89 mL, *p* = 0.008) and the amount of hemofiltration was consecutively higher (4388.88 ± 2168.20 mL vs. 2274.02 ± 2331.49 mL, *p* = 0.013) in patients with postoperative AKI.

Patients with AKI had a more complicated postoperative course with a prolonged ICU stay and a higher incidence of all reported postoperative morbidities, including pulmonary, neurological, bleeding, infectious and hemodynamic complications (Table 3).

Moreover, mortality after 7 and 30 days was significantly higher in patients with renal dysfunction (7d-mortality: 4/13 vs. 0/88, *p* < 0.001, 30-d-mortality: 9/13 vs. 1/88, *p* < 0.001).

**Figure 2:**
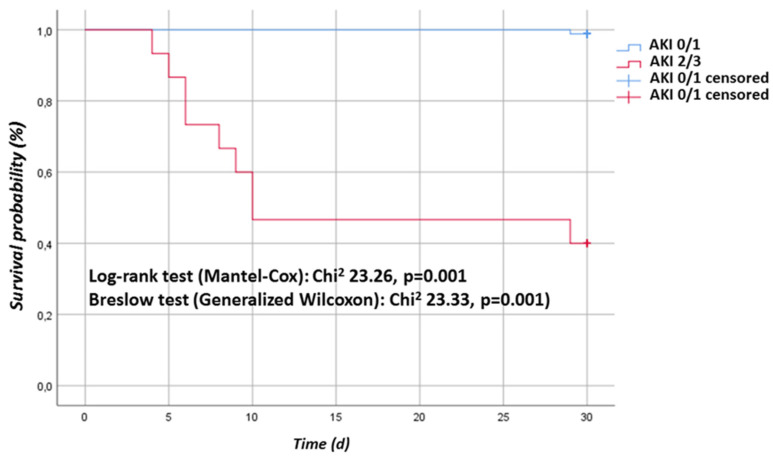
Kaplan-Meier analysis: Kaplan-Meier survival curve. AKI 0/1 group vs. AKI 2/3 group.

In addition, we performed a Kaplan-Meier survival analysis based on AKI staging. Severe AKI was significantly associated with 30 d mortality (Log-rank test (Mantel-Cox): Chi2 23.26, *p* = 0.001, Breslow test (Generalized Wilcoxon): Chi2 23.33, *p* = 0.001, Figure 2). Cox proportional hazard ratio analysis based on AKI staging showed that severe AKI stage 2 or 3 was significantly associated with 30 d mortality (HR 0.013 (95% CI 0.002–0.104); *p* = 0.001).

### 3.2. Perioperative Course of Biomarkers

**Figure 3:**
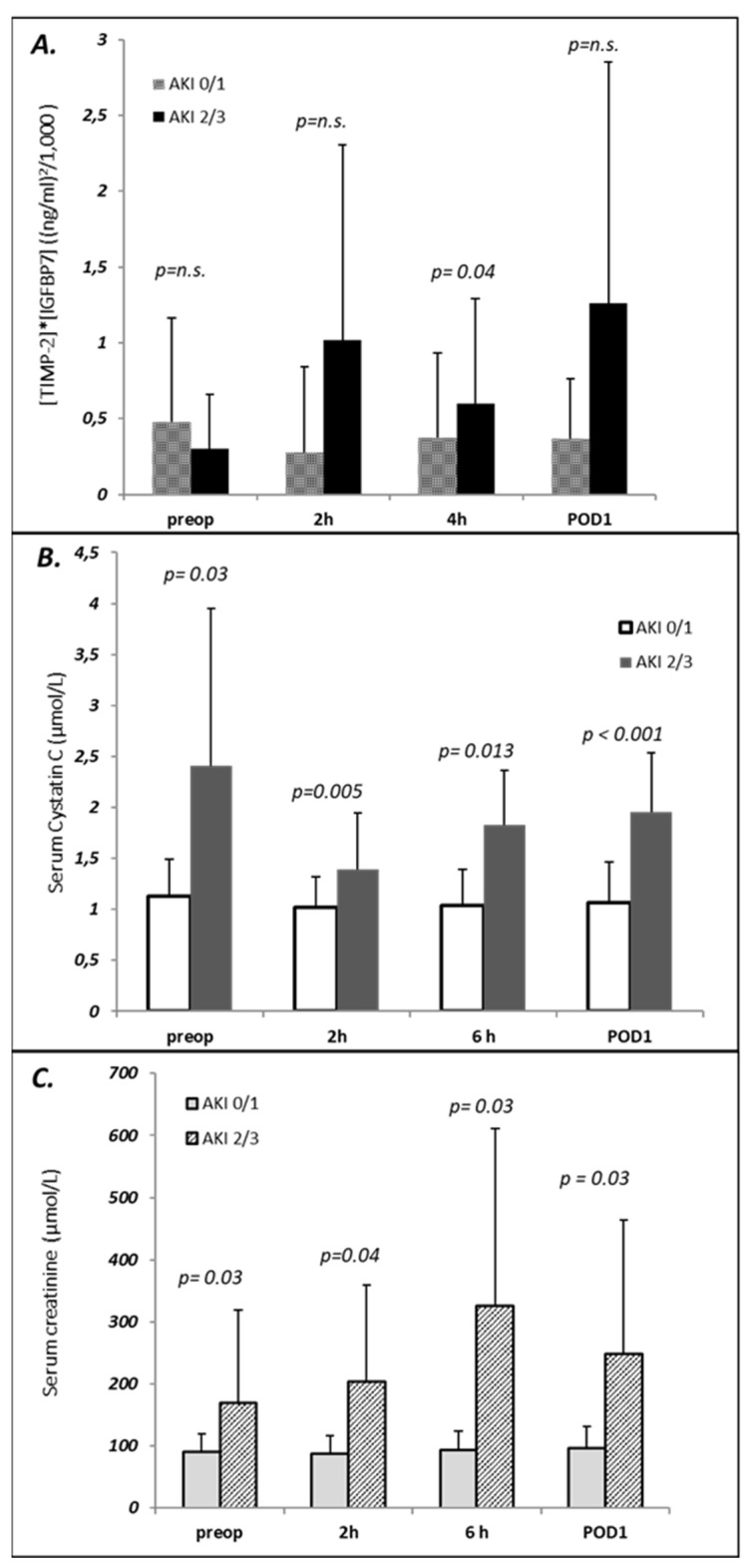
Perioperative course of biomarkers in patients with AKI ≥ 2 compared to patients with AKI ≤ 1. (**A**) Urinary [TIMP-2]*[IGFBP7]; (**B**) Serum Cystatin; (**C**) Serum creatinine.

The postoperative course of serum creatinine, Cystatin C and urinary [TIMP-2]*[IGFBP7] for patients with AKI stage 2/3 and those with no or mild AKI is illustrated in Figure 3.

Whereas preoperative serum creatinine (169.73 ± 148.97 μmol/L vs. 89.74 ± 30.04 μmol/L, *p* = 0.027) as well as Cystatin C (2.41 ± 1.54 mg/L vs. 1.13 ± 0.35 mg/L, *p* = 0.029) were higher in the AKI group, [TIMP-2]*[IGFBP7] before surgery did not differ between groups (0.29 ± 0.35 vs. 0.48 ± 0.69, *p* = n.s.).

No significant rise in Cystatin C, serum creatinine or urinary [TIMP-2]*[IGFBP7] was observed in patients with AKI 0/1 at any time indicating that TAS with MHCA per se has no influence on the investigated G1 cell cycle arrest biomarkers or Cystatin C.

In patients developing AKI 2/3 within the first 48 postoperative hours, [TIMP-2]*[IGFBP7] increased significantly four hours after surgery (0.6 ± 0.69 vs. 0.37± 0.56, *p* = 0.03) but not 2 h after surgery (1.2 ±1.29 vs. 0.28 ± 0.56, *p* = n.s.).

### 3.3. Prediction of AKI with Biomarkers

Using ROC-analyses, preoperative Cystatin C showed a sensitivity of 79.0% and a specificity of 76.8% for predicting AKI any stage (AUC 0.828, *p* < 0.001), being superior to the prognostic performance of serum creatinine (AUC 0.686, *p* = 0.002, sens. 42.9%, spec. 93.2%) in deLong testing (*p* = 0.0211, Table 4). In addition, the predictive capacity of preoperative Cystatin C for AKI stage 2/3, AKI with need for RRT and 30-d-mortality was very strong (AKI 2–3: AUC 0.933, *p* < 0.001, RRT: AUC 0.919, *p* < 0.001; 30-d-mortality: AUC 0.887, *p* < 0.001). Whereas [TIMP-2]*[IGFBP7] before surgery and 2 h after surgery showed a low predictive capacity, [TIMP-2]*[IGFBP7] 4 h after surgery was able to predict postoperative AKI (AUC 0.724, *p* = 0.020). Serum creatinine at all time points was able to predict AKI any stage, AKI 2/3 and AKI with need for RRT.

## 4. Discussion

Moderate hypothermic circulatory arrest (MHCA) for cerebral protection during aortic arch operations was first introduced in 1976 and is widely used presently for neuroprotection during complex congenital heart surgery and aortic surgery. Although MHCA has proven to be an effective technique for protecting various organs from ischemic damage as a result of circulatory arrest, AKI after MHCA has a reported incidence of 5% to 50% depending on definition and patient cohort [1,2,3,4,5].

Incidence of AKI all stages was 26.5% (27/101) in our study. Looking at the current literature on AKI in the general critical care population as well as in patients after cardiac surgery, prevalence and incidence of AKI vary significantly [1,2,3,4,5]. There are many possible reasons explaining this observation. First, varying incidence and prevalence of AKI may be the result of a different definition employed. A recently published study showed that the incidence of AKI in critically ill patients varied according to the criteria used [15]. We used the KDIGO definition in our study. However, even if other studies used the same definition, incidence of AKI may still vary, as multiple ways of applying the KDIGO criteria exist. A recently published study demonstrated that incidence of AKI varied from 28–75%, depending on the method used for applying the KDIGO criteria [16].

Indication for dialysis was not uniformly defined in most published studies, nor in our study, and was frequently dependent on the discretion of the treating nephrologists or intensivist. Therefore, it is possible that other studies and our analyses might not be comparable in defining AKI stage 3. In addition, the previous findings of a high incidence of AKI among patients undergoing thoracic aortic surgery may have been confounded by patient selection.

At first glance, it might be surprising that length of surgery and cardiopulmonary bypass (CPB) time were significantly longer in the AKI group, but length of MHCA did not differ. Whereas the majority of studies are in accordance with our finding in showing an independent association of CBP time and AKI, some studies have revealed body ischemic time as a risk factor for post-operative AKI [17].

The inconsistency of these results may be due to the different populations and inconsistent definitions and diagnoses of AKI. The etiology of cardiac surgery-associated AKI is complex, but there is strong evidence from studies in experimental animals, clinical observations and computational models, that medullary ischemia and hypoxia occurs during CPB. It is hypothesized that MHCA could significantly extend the CPB time, which is likely to be counterbalanced by the protective effects of DHCA on renal function.

AKI is associated with an increased morbidity and mortality which is proportional to the severity of AKI. Patients requiring RRT have a mortality as high as 64%. Therefore, there is a clear demand for early detection or even prognostication of AKI. Traditionally, serum creatinine and urine output are the two major clinical indicators of AKI. However, several other factors, such as muscle volume and diuretic use, also impact on these indicators: serum creatinine levels increase along with a decrease in glomerular filtration rate, but not in the early stage of AKI, which is mainly caused by tubular injury and necrosis [6]. Serum creatinine levels do not reflect renal damage in time for an early diagnosis because an increase in serum creatinine occurs from several hours to as late as two or three days after the onset of AKI. Thus, the value of serum creatinine for early diagnosis of AKI is very limited [6]. Although urine output is a good indicator of AKI, renal damage without oliguria renders this criterion less reliable. Hence, the identification of a more suitable biomarker has become a central issue to improve early diagnosis and management of AKI. Early treatment may be able to improve the prognosis or even reverse the acute renal damage in AKI and reduce AKI-associated mortality [7]. Various biomarkers of AKI have been evaluated in recent years, including Cystatin C and [TIMP-2]*[IGFBP7] [8,9].

Cystatin C is a non-glycosylated, 13.3-kDa protein belonging to the cystatin protease inhibitors [9]. After glomerular filtration, it is fully catabolized in the proximal renal tubule and is not passed back into the blood. It might be a promising alternative to serum creatinine in estimation of GFR. A recently published prospective study with patients, undergoing cardiac surgery serum, as well as urinary Cys-C within the first 6 h after surgery, was able to predict AKI [18]. A meta-analysis including 30 prospective cohort studies (involving 4247 adults from 15 countries, 982 patients with AKI) showed a high predictive power of serum Cystatin C for all-cause AKI with an AUC of 0.89 [19]. Other studies confirm the predictive capacity of Cystatin C [20,21]. Our analysis is in accordance with these findings, showing that Cystatin C represents a sensitive and specific biomarker to predict AKI in patients undergoing thoracic surgery with MHCA. In addition, we were able to prove a prognostic value for preoperative serum Cystatin C predicting postoperative AKI.

In recent years, a new AKI biomarker system, a composite of the two independent proteins metalloproteinase inhibitor 2 (TIMP2) and the IGF-binding protein 7 (IGFBP7), in urine samples has been introduced and has been widely accepted on the basis of a large number of clinical trials [9,22,23]. Multicenter studies revealed TIMP-2 and IGFBP7 to have a better performance than any other existing marker for prediction of AKI [22,23].

As etiologies and pathophysiology of AKI differ substantially between patient populations, biomarkers of AKI may not be reliable in all fields. The performance of every biomarker in different patient cohorts and clinical settings may vary significantly requiring careful and specific evaluation of the marker in different scenarios. This also applies for G1 cell cycle arrest marker: IGFBP7 is superior to TIMP-2 in surgical patients whereas TIMP-2 is better for prediction of septic AKI, demonstrating that these two biomarkers are involved in slightly different pathways. Therefore, it is not clear whether the excellent results of the Discovery, Sapphire and Opal study can be transferred to patients undergoing TAS with MHCA.

Accordingly, although the majority of studies reported excellent predictive capacity of this biomarker in critically ill patients, there were also studies reporting negative results for [TIMP-2]*[IGFBP7]. A recently published single-center, prospective observational study enrolling 111 decompensated cirrhotic patients without AKI at the time of admission showed plasma Cystatin C and urine NGAL but not urine [TIMP-2]*[IGFBP7] to be potentially useful biomarkers for early sensitive detection of AKI [24]. The production of [TIMP-2]*[IGFBP7] from damaged hepatocytes in chronic liver disease patients might be one possible mechanism explaining the negative results. Studies on patients after cardiac surgery also showed divergent results for [TIMP-2]*[IGFBP7] [10,11,25,26]. In summary, Cystatin C represents a very sensitive and specific biomarker to predict AKI in patients under-going thoracic surgery with MHCA even before surgery, whereas the predictive capacity of [TIMP-2]*[IGFBP7] is only moderate. A single preoperative measurement of Cystatin C allows the identification of patients who will develop postoperative AKI, especially those with severe AKI.

The excellent predictive capacity of postoperative serum creatinine might be unexpected but is explainable by the fact that AKI occurred very early after surgery, and serum creatinine is one of two parameters to define and classify AKI. However, the predictive capacity of Cystatin in the preoperative setting was significantly superior to serum creatinine. Whereas serum creatinine represents a diagnostic marker for AKI, Cystatin C allows true preoperative identification and prognostication of AKI.

Difficulties in prediction and early identification of AKI have hindered the ability to develop preventive and therapeutic interventions. If AKI is recognized early or even predicted preoperatively by cystatin C, nephroprotective measures could be considered to reduce exposure to renal insults and potentially avoid the development of higher stage AKI. Although the discussion about preventive or therapeutic interventions in critically ill patients with AKI is controversial, there are some strategies that may be beneficial in the perioperative setting: if AKI is predicted preoperatively, preventive strategies could include a delay of surgery if elective, with optimization of renal function including the optimization of fluid balance and hemodynamics, as well as a medication review with avoidance of nephrotoxic drugs, as this can reduce the incidence and severity of AKI and improve long-term outcomes [27]. Accordingly, a nephrologist should be consulted, as a delayed consultation was associated with higher mortality and increased dialysis dependence rates in critically ill AKI patients at hospital discharge [28]. Preoperative remote ischemic preconditioning significantly reduced the rate of AKI and use of RRT in patients undergoing cardiac surgery in a recently published multicenter randomized trial [29]. In addition, earlier postoperative commencement of RRT in critically ill patients with incipient AKI may be beneficial with a reduction in mortality, in particular for patients after cardiac surgery [30,31].Therefore, a bundle of interventions should be initiated if high preoperative Cystatin C levels predict postoperative AKI including consultation with a nephrologist, extensive chart review and stopping of all nephrotoxic agents, perioperative goal-directed hemodynamic management, delay, if possible, of any exposure to contrast agent or even surgical intervention, discussion of early RRT in case of AKI, and delay of transferring the patient from the ICU to the normal ward. To our knowledge, currently there are only studies published on preventive or therapeutic approaches based on [TIMP2]*[IGFBP7] but not Cystatin. Therefore, prospective intervention trials are necessary to prove if early risk-assessment with Cystatin C and risk-adjusted management can reduce AKI incidence, severity or progression and associated mortality after thoracic aortic surgery with moderate hypothermic circulatory arrest.

## 5. Limitations

Due to the relatively small sample size, further studies are necessary to confirm the excellent predictive capacity of Cystatin C and to investigate whether preventive interventions based on this biomarker can help to reduce postoperative AKI as well as associated morbidity and mortality. The limited sample size of our study did not allow for extensive adjustments in multivariate modelling, e.g., age, sex, comorbidities. Therefore, our results need to be interpreted with caution and warrant confirmation in independent, preferably larger studies. However, large validation studies of the Nephrocheck test showed that urine [IGFBP7]*[TIMP-2] values were not elevated in patients with stable chronic morbidities such as diabetes mellitus, congestive heart failure and CKD who did not have AKI and were not affected by sex or age [26]. Other studies in cardiac surgery showed that there is no independent association between CBP time or other operative variables and [IGFBP7]*[TIMP-2] values.

CysC is produced at a constant rate by all nucleated cells and its concentration is not influenced by age, sex, height, and body composition. Concerning preoperative variables, patients with postoperative AKI were older and suffered from a higher NYHA class as well as higher prevalence of atrial fibrillation in our study. Many studies have demonstrated the prognostic value of CysC for AKI in the setting of acute heart failure as well as in stable chronic heart failure, unaffected by cardiac function or heart rhythm [5,6,7]. However, inflammatory status, hyperthyroidism, glucocorticoids use, and current smoking status can influence cys-C levels. Lack of adjustment of these residual or unmeasured confounding factors may have overestimated the risk estimate. Smoking status as well as CRP-levels did not differ between AKI- und non-AKI-patients, but we did not assess the thyroid status or glucocorticoids use in our analysis.

In the current study, urinary creatinine was not measured to estimate the true creatinine clearance for these patients. There are three major errors that can limit the accuracy of the creatinine clearance as an estimate of GFR: errors in urine collection, increases in creatinine secretion and extra renal creatinine degradation as the GFR falls. Because of these limitations, we did not collect 24 h urine to calculate creatinine clearance but used the Cockcroft-Gault formula to estimate GFR.

## Figures and Tables

**Table 1:** Classification criteria for staging of AKI.

AKI KDIGO Stage	Number	Classification Criterion for AKI Stage	
		Increase of Serum Creatinine	Decrease of Diuresis or RRT
1	14 (13.9%)	8	6
2	3 (3.0%)	2	1
3	10 (9.9%)	1	9
All (1–3)	27 (26.7%)	11	16

AKI = acute kidney injury, KDIGO = Kidney Disease: Improving Global Outcomes, RRT = renal replacement therapy.

**Table 2:** Patient characteristics of patients with AKI KDIGO ≥ 2 compared to patients with no or mild AKI (KDIGO 0–1).

	AKI 0–1 (*n* = 88)	AKI 2–3 (*n* = 13)	*p*-Value
Age (years)	68.51 ± 11.46	72.72 ± 6.19	0.044
Female Sex (*n*,%)	60 (68.2)	8 (61.5)	n.s.
Weight (kg)	82.51 ± 16.02	8.67 ± 17.25	n.s.
Height (cm)	174.20 ± 9.01	171.73 ± 9.13	n.s.
BMI(kg/m^2^)	27.09 ± 4.28	27.20 ± 4.82	n.s.
ES I log	19.89 ± 14.66	29.68 ± 19.98	0.026
ES II (%)	5.73 ± 5.25	16.29 ± 11.49	0.003
STS (%)	3.38 ± 3.34	5.44 ± 4.64	n.s.
EF (%)	57.89 ± 13.71	53.69 ± 13.49	n.s.
Acute MI (*n*,%)	5 (56.8)	2 (15.4)	n.s.
NYHA ≥ III (*n*,%)	10 (11.4)	6 (46.2)	0.036
COPD (*n*,%)	8 (9.1)	1(7.7)	n.s.
aHTN (*n*,%)	63 (71.6)	14	n.s.
HLP (*n*, %)	41(46.6)	9 (69.2)	n.s.
pHTN (*n*,%)	2 (2.2)	1 (7.7)	n.s.
Afib (*n*,%)	11(12.5)	6 (46.2)	0.017
PAD (*n*,%)	11 (12.5)	3 (23.1)	n.s.
CAD (*n*,%)	44 (50.0)	9 (69.2)	n.s.
Redo (*n*,%)	11 (12.5)	3 (23.1)	n.s.
s/p Stroke (*n*,%)	10 (11.4)	1 (7.7)	n.s.
Endocarditis (*n*,%)	2 (2.3)	0 (0)	n.s
Smoking (*n*,%)	18 (20.5)	3 (23.1)	n.s.
CKD (*n*,%)	0 (0)	5 (38.5)	0.001
Urgent/Emergency Surgery (*n*,%)	15 (17.0)	5 (38.5)	0.02
Duration Surgery (min)	297.77 ± 93.29	397.20 ± 139.04	0.017
Duration CPB (min)	170.57 ± 51.05	230.21 ± 68.29	0.007
Cross Clamp Time (min)	177.22 ± 42.93	151.50 ± 62.38	0.066
CA Duration (min)	24.70 ± 31.97	33.14 ± 31.77	*n*.s.
RPBC intraop. (n, %)	1.61 ± 2.74	4.27 ± 3.71	0.001
Dosage of epinephrine (mg/h)	0.21 ± 0.43	0.60 ± 0.51	0.011
Diuresis intraop. (mL)	989.82 ± 788.89	557.14 ± 605.06	0.008
HF intraop (mL)	2274.02 ± 2331.49	4388.88 ± 2168.20	0.013
Aneurysm size (mm)	52.51 ± 7.71	44.73 ± 9.79	0.004
Marfan (*n*,%)	2 (2.3)	0 (0)	n.s.
Supra-coronary Asc.-Replacement (*n*, %)	83 (94.3)	12 (92.3)	n.s.
Hemiarch replacement (*n*,%)	30 (34.1)	4 (30.8)	n.s.
Total arch replacement (*n*, %)	5 (5.7)	2 (15.4)	n.s.
Aortic root surgery (*n*,%)	14 (15.9)	4 (30.8)	n.s.
David-Procedure (*n*,%)	9 (10.2)	1 (7.7)	n.s.
Elephant trunk (*n*,%)	1 (1.1)	2 (15.4)	n.s.
CABG (*n*, %)	37 (42.0)	7 (53.8)	n.s.
AVR (*n*,%)	44 (50.0)	7 (53.8)	n.s.
MVS (*n*,%)	3 (3.4)	3 (23.1)	0.038
TVS (*n*,%)	0 (0)	2 (15.4)	0.02

aHTN = arterial hypertension, Afib = atrial fibrillation, AVR = Aortic valve replacement, BMI = Body mass index, CA = Cardiac arrest, CAD = coronary artery disease, CABG = coronary artery bypass grafting, CKD = chronic kidney disease, COPD = chronic obstructive pulmonary disease, ES = EuroScore, HF = hemofiltration, MVS = mitral valve surgery, PAD = peripheral artery disease, PHT = pulmonary hypertension, STS = Society of Thoracic Surgeons, TVS = tricuspid valve surgery.

**Table 3:** Postoperative outcome of patients with AKI KDIGO ≥ 2 compared to patients with no or mild AKI (KDIGO 0–1).

Outcome	AKI 0–1 (*n* = 88)	AKI 2–3 (*n* = 13)	*p*-Value
Reintubation (*n*,%)	7 (8.0)	7 (53.8)	0.001
PDT (*n*,%)	3 (3.4)	6 (61.2)	0.001
Neurological complication (*n*,%)	6 (6.8)	6 (61.2)	0.001
CPR (*n*,%)	0 (0)	3 (21.4)	<0.001
Myocardial infarction (*n*,%)	0 (0)	2 (14.3)	0.002
Pneumonia (*n*,%)	4 (4.5)	7 (53.8)	<0.001
Sepsis (*n*,%)	2 (2.3)	7 (53.8)	<0.001
Re-exploration for bleeding (*n*,%)	3 (3.4)	9 (64.3)	<0.001
Infections (*n* %)	2 (2.)	3 (21.4)	0.012
DSWI (*n*,%)	0 (0)	1 (7.1)	0.048
7 d mortality (*n*,%)	0 (0)	4 (30.8)	<0.001
30 d mortality (*n*,%)	1 (1.1)	9 (69.2)	<0.001

CPR = cardiopulmonary resuscitation, DSW I = deep sternal wound infections, PDT = percutaneous dilatational tracheostomy.

**Table 4:** Prediction of postoperative AKI with Biomarker.

Variable	AUC (95% CI)	SE	*p*-Value	Cut-Off	Sensitivity	Specificity
**Prediction of AKI any stage**						
*Cystatin C preop.*						
preoperative	0.822 (0.710–0.935)	0.057	0.000	1.19	0.79	0.75
2 h postoperative	0.713 (0.594–0.869)	0.070	0.0028	1.22	0.88	0.56
6 h postoperative	0.872 (0.779–0.964)	0.047	<0.001	1.09	0.94	0.73
POD 1	0.956 (0.841–0.970)	0.033	<0.001	1.09	1.00	0.71
*Serum-Creatinine*						
preoperative	0.679 (0.530–0.828)	0.076	0.022	123.5	0.47	0.96
2 h postoperative	0.822 (0.729–0.915)	0.047	<0.001	80.5	0.45	0.95
6 h postoperative	0.894 (0.783–1.000)	0.0562	<0.001	121.5	76.92	90.38
POD 1	0.871 (0.779–0.962)	0.046	<0.001	96.5	0.95	0.67
[TIMP-2]*[IGFBP7]						
preoperative	0.381 (0.212–0.549)	0.086	0.165	0.205	0.5	0.442
2 h postoperative	0.534 (0.336–0.733)	0.101	0.734	0.19	0.417	0.692
4 h postoperative	0.692 (0.537–0.848)	0.079	0.015	0.205	0.583	0.712
POD 1	0.635 (0.445–0.824)	0.097	0.164	0.385	0.583	0.75
**Prediction of AKI stage 2–3**						
*Cystatin C preop.*						
preoperative	0.930 (0.857–1.000)	0.037	0.000	1.53	0.8	0.95
2 h postoperative	0.731 (0.529–0.934)	0.103	0.0438	1.27	0.87	0.57
6 h postoperative	0.907 (0.806–1.000)	0.0516	0.0005	1.42	0.89	0.86
POD 1	0.909 (0.830–0.989)	0.0406	0.004	1.33	1.00	0.78
*Serum-Creatinine*						
preoperative	0.813 (0.643–0.982)	0.087	0.002	130.0	0.7	0.97
2 h postoperative	0.919 (0.857–0.983)	0.032	<0.001	98.5	1.00	0.76
6 h postoperative	0.975 (0.925–1.000)	0.026	<0.001	120.5	1.00	0.83
POD 1	0.878 (0.714–1.000)	0.084	<0.001	159.5	0.98	0.80
[TIMP-2]*[IGFBP7]						
preoperative	0.409 (0.270–0.548)	0.071	0.198	0.17	0.75	0.397
2 h postoperative	0.740 (0.429–1.052)	0.159	0.131	0.265	0.75	0.794
4 h postoperative	0.724 (0.535–0.913)	0.096	0.020	0.215	1.00	0.556
POD 1	0.544 (0.233–0.854)	0.159	0.783	0.165	0.75	0.413
**Prediction of RRT**						
*Cystatin C*						
preoperative	0.944 (0.853–1.036)	0.047	0.000	1.53	0.86	0.92
2 h postoperative	0.745 (0.537–0.953)	0.106	0.033	1.46	0.57	0.95
6 h postoperative	0.891 (0.774–1.000)	0.059	0.001	1.42	0.84	0.87
POD 1	0.934 (0.866–1.000)	0.035	<0.001	1.33	1.00	0.77
Serum-Creatinine						
preoperative	0.861 (0.623–1.099)	0.121	0.003	130.0	0.71	0.94
2 h postoperative	0.882 (0.809–0.954)	0.0367	<0.001	104.0	1.00	0.76
6 h postoperative	0.909 (0.807–1.000)	0.0517	0.0175	121.5	1.00	0.81
POD 1	0.788 (0.572–1.000)	0.110	0.01	121.5	0.86	0.76
[TIMP-2]*[IGFBP7]						
preoperative	0.378 (0.204–0.552)	0.89	0.168	0.17	0.667	0.391
2 h postoperative	0.667 (0.169–1.164)	0.254	0.511	1.44	0.667	0.969
4 h postoperative	0.643 (0.241–1.045)	0.205	0.485	0.65	0.667	0.79
POD 1	0.404 (0.078–0.885)	0.246	0.695	1.28	0.478	0.984

AKI = acute kidney injury, AUC = area under the curve, CI = confidence interval, POD = postoperative day, RRT = renal replacement therapy, SE = standard error.

## Data Availability

The data presented in this study are available on request from the corresponding author. The data are not publicly available due to legal or privacy issues.
